# Identification of an *in vitro* artemisinin-resistant *Plasmodium falciparum* kelch13 R515K mutant parasite in Senegal

**DOI:** 10.3389/fpara.2023.1076759

**Published:** 2023-04-28

**Authors:** Seynabou D. Sene, Mariama N. Pouye, Rafael Miyazawa Martins, Fatoumata Diallo, Khadidiatou Mangou, Amy K. Bei, Alioune Barry, Oumar Faye, Oumar Ndiaye, Ousmane Faye, Amadou A. Sall, Jose-Juan Lopez-Rubio, Alassane Mbengue

**Affiliations:** ^1^ G4-Malaria Experimental Genetic Approaches and Vaccines, Pôle Immunophysiopathologie et Maladies Infectieuses, Institut Pasteur de Dakar, Dakar, Senegal; ^2^ Laboratory of Pathogen-Host Interactions (LPHI), Unité Mixte de Recherche 5235 (UMR5235), Centre national de la recherche scientifique (CNRS), University of Montpellier, Institut National de la Santé Et de la Recherche Médicale (INSERM), Montpellier, France; ^3^ Department of Epidemiology of Microbial Diseases, Yale School of Public Health, New Haven, CT, United States; ^4^ Epidemiology, Clinical Research and Data Science Unit, Institut Pasteur de Dakar, Dakar, Senegal; ^5^ Arboviruses and Haemorrhagic Fever Viruses Unit, Virology Department, Institut Pasteur de Dakar, Dakar, Senegal

**Keywords:** *P. falciparum*, artemisinin-resistance PfKelch13, genetic variation, CRISPR-Cas9, genomic surveillance system, Senegal

## Abstract

The emergence of artemisinin partial resistance (ART-r) in *Plasmodium falciparum* malaria parasites has substantially compromised the efficacy of antimalarial treatments across southeast Asia (SE Asia). The spread of ART-r within the African continent could jeopardize past progress made in reducing worldwide malaria burden. A clinical index malaria case was identified in Kaolack, Senegal with persistent fever after complete artesunate-amodiaquine (ASAQ) treatment. Fifteen malaria-infected blood samples were collected by Institut Pasteur Dakar’s Senegalese sentinel surveillance system, from different healthcare centers surrounding the index case. We have identified one *Plasmodium falciparum* clinical isolate carrying R515K mutation in the artemisinin resistance gene PfKelch13. CRISPR-Cas9 genome editing was carried out and transgenic Pf3D7Pfkelch13^R515K^ was tested for *in vitro* standard Ring-stage Survival Assay *(RSA* ^0-3hpi^). Gene editing has confirmed that *PfKelch13^R515K^* drove increased *in vitro* RSA^0-3hpi^ value. In this article, we report the functional significance of PfKelch13^R515K^ mutation in an African context.

## Introduction

Artemisinin (ART) and its derivatives are the cornerstone of malaria case management for which no replacement is currently available on the market. The introduction of WHO recommended Artemisinin-based Combination Therapy (ACTs) as first-line treatment for uncomplicated malaria cases has partially contributed to the notable reduction of global malaria (WHO, 2015; World Health Organization. (2016)). ART has a short life and a rapid killing action for *Plasmodium* asexual blood stage parasites (Intharabut et al., 2019). Upon treatment, the rings are quickly eliminated and infected erythrocytes removed from the bloodstream, preventing sequestration (Mbengue et al., 2012). ART-r causes delayed parasite clearance upon 3 days of ACT treatment and increased *in vitro* RSA^0-3hpi^ value. The parasite gene *PfKelch13* (Pf3D7_1343700) discovered through a candidate gene approach is a primary marker of ART-r (Noedl et al., 2008; Ariey et al., 2014; Miotto et al., 2015). PfKelch13 mutations associated with ART-r were characterized in South East Asia (SEA) *Plasmodium falciparum* clinical isolates (Mbengue et al., 2015; Miotto et al., 2015; Mok et al., 2015; Tun et al., 2015). Since 2008, ART-r has been reported in India and a province in China (Huang et al., 2015; Das et al., 2018). In Africa where the malaria burden remains high, ART-r is a serious threat that has been documented in Rwanda and Uganda (Uwimana et al., 2020; Balikagala et al., 2021). As for chloroquine, ART-r will, if not contained, be a threat to malaria disease control plans in Sub-Saharan Africa (Trape et al., 1998; Lubell et al., 2014; Mukherjee et al., 2017; Sutherland et al., 2017). Clustered Regularly Interspaced Palindromic Repeat-Cas9 (CRISPR-Cas9) genome editing has been successfully applied in *P. falciparum* to confirm *in vitro the* function of key polymorphisms or alleles associated with resistance to antimalarials (Ghorbal et al., 2014; Lee and Fidock, 2014; Mbengue et al., 2015).

We found in Senegal, *P. falciparum* isolates with PfKelch13^R515K^ an ART-r associated variant found in SEA upon ACT treatment (WWARN K13 Genotype-Phenotype Study Group, 2019). Transgenic lines were generated using CRISPR-Cas9 technology in the African background laboratory-adapted strain *P. falciparum 3D7*. Phenotypic assays of the transgenic Pf3D7Kelch13^R515K^ line were performed following established RSA^0-3hpi^ protocol (Witkowski et al., 2013).

## Material and methods

### Sample collection and plasmodium gene amplification

**Figure 1:**
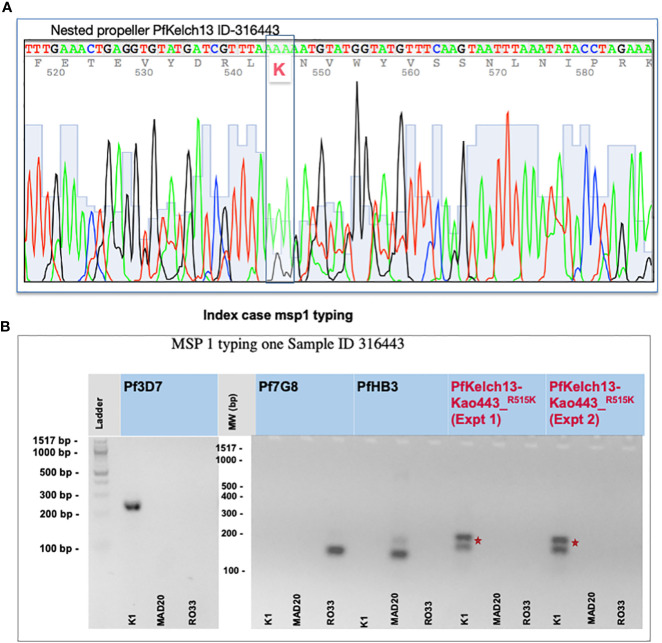
Identification of the R515K mutation and complexity of malaria infection. **(A)** PfKelch13 amplification from the original DNA batch was done. A major peak of Arginine (R codon AAA) and a minor peak of K (AGA) were detected at position 515 in the chromatogram indicating a mixed multi-clonal malaria parasite infection. **(B)** msp1 typing shows multiple clonal falciparum infections in the index case (ID 316443). gDNA from 3D7, Pf7G8, and PfHB3 strains were used as monogenic control for the complexity of the infected rate. Primers are provided in **Supplementary Table 1**. Experiments-1 and 2 represent biological replicates. *Shows the position of the nucleotide change before and after crispr cas.

Blood samples were collected by the IPD-4S in November 2018 (Surveillance des fièvres, 2015). The IPD-4S network is an important surveillance system initially built to strengthen influenza sentinel surveillance in partnership with the Senegalese Ministry of Health since 2012. This network of researchers, medical doctors, and nurses is implemented in all 14 regions of Senegal. Among its top priorities, the IPD-4S network also helps to routinely identify unusual health events to provide a rapid and appropriate medical response to the communities. Between 2017 and 2018 a surveillance program in response to a Dengue outbreak was carried out (Dieng et al., 2021). For this investigation, all declared PfRDT (SD Bioline malaria AG P.F) positive samples from the surrounding index case (ID 316443) area were collected from Ndoffane and other health care centers from the same region Kaolack as well as in the neighborhood region Diourbel. Malaria-positive samples were collected following the IPD-4S infection transmission control plan. Fifteen malaria-positive samples were collected, and 2 ml of venous blood was then shipped at 4°C to IPD the following day. Clinical information of the 18-year-old girl at day 9 post ACT treatment (ID 316443) is presented in **Table 1b**. DNA from the erythrocyte pellet was extracted using Quick-gDNA Blood MiniPrep kit from ZYMO research following the manufacturer’s instructions. Nested primers were designed to amplify the propeller domain of *Kelch13*. Full-length PfKelch13 was also amplified from biological replicates. Chromatograms of all PCR products were analyzed and the multiplicity of infection rate was determined using msp1 and msp2 typing protocol (**Figure 1** and **Supplementary Figure 1**). Human malaria genius typing was done using a light cycler and LightMix modular Plasmodium genus (Malaria) Cat # 53-0694-96 and 40-0694-24 respectively (TIB BioMol). All primers are listed in Supplementary files. (**Supplementary Tables 2A, B**).

**Table 1:** First appearance of PKelch13^R515K in^ Kaolack, Central Senegal.

a		b	
Sample ID	PfKelch 13 SNPs	Clinical symptoms of the 18 years old infected patient AT Day 9 post ASAQ treatment	
316441	WT		
316443	R515K	IPD Patient Number	316443
316453	WT	Age	18
316501	WT	Sex	female
316524	WT	Axillary temperature	37°C
316526	WT	Vomiting	Yes
316527	WT	Headache	Yes
316528	WT	asthenia	Yes
316531	WT	eyes pain	Yes
316550	WT	muscle aches	No
316598	WT	Rashes	No
316510	WT	Meningo-Encephalitis	No
316548	WT	Abdominal pains	No
316649	WT	PfRDT (SD Bioline malaria AG P.F)	Positive
316373	WT		

a) Identification of PfKelch13 single Nucleotide polymorphism. Nested PCR of the parasite ART-r gene marker Pfklech13 was done using high-fidelity enzymes. R515K variant was found in clinical isolates collected from the index case (ID 316443). A total of 14 malaria-infected blood samples collected the same day in the neighborhood health care centers show wild-type PfKelch13. The clinical symptoms of the 18 year old female patient (ID: 316443) are described in b). This patient has received ASAQ treatment and came back to the health care center with fever persistence 6 days after completing ASAQ treatment i.e., 9 days from her first visit.

### Plasmid construct

The two-plasmid approach was employed to express Cas9, sgRNA, and a donor template (Ghorbal et al., 2014). SpCas9 was delivered on the pUF1 plasmid, which also contains a yeast dihydroorotate dehydrogenase (ydhodh) expression cassette which confers resistance to PfDHODH inhibitors such as DSM1. The sgRNA and the donor DNA template for homologous recombination repair were placed in the same plasmid pL7 (here pL7-238, have already cloned with the seed so we only need to clone the donor DNA). pL7 also expresses human dihydrofolate reductase (hdhfr) allowing positive selection with WR99210. The donor DNA to be used as a template to repair the double-strand breaks (DSBs) generated by Cas9 was designed from the *kelch13* gene of *Plasmodium falciparum* 3D7 (PlasmoDB ID PF3D7_1343700). Donor’s DNA includes the homologous regions (HR1 and HR2) flanking the region of interest (ROI). The ROI carries the desired mutation and an additional modification (defined here as shield mutations) at the Cas9-target site. The shield’s mutations are silent but abolish recognition by Cas9, thereby protecting the modified locus from repeated cleavage. Additional silent mutations spanning the gap between the shield mutations and the desired modification can be introduced to help drive the repair event beyond the mutation-of-interest. Homology regions with plasmid and restriction sites were added for cloning. Donor DNA (synthetic DNA by Integrated DNA technologies) was cloned in pL7 plasmid at *SpeI-AflII* sites using Infusion cloning technology (Takara-Bio) (**Supplementary Figure 2A**).

### Parasite culture and transfection

*P. falciparum* asexual blood-stage parasites 3D7 wild-type were cultured in A^+^ human red blood cells (RBCs) in RPMI-1640 culture medium containing 25 mM Hepes + l-glutamine, supplemented with 10% Albumax II (Gibco Life Technologies), Human Sera (HS), hypoxanthine (C.C.Pro GmbH) and gentamicin (Sigma). Parasites were maintained at 37°C in 5% O_2_, 5% CO_2_, and 90% N_2_. Cultures were monitored by blood smears fixed in methanol, stained with Giemsa, and viewed by light microscopy. Synchronous cultures were obtained by sorbitol treatment. Prior to transfection, 50 μg of each plasmid circular DNA (pUF1-Cas9 and recombinant plasmid pL7) were ethanol-precipitated and resuspended in 30 μl of Tris-EDTA. The DNA precipitated plasmids were co-transfected into 100 μl rings stages parasites at 4.87% parasitemia and 270 μl cytomix, by electroporation using the Bio-Rad GenePulse Xcell™ electroporator, at 310 V, with a resistance of 950 μF and a transfection time of less than 10s.

Drug pressure was applied 15–20 h after transfection: WR99210 for pL7-238_Insert was used at 2.5 nM and DMS1 for pUF1-Cas9 was used at 1.5 μM. Media and drugs were renewed every 24 h for the first 5 days, then every other day for a week, and twice a week until parasites are visually detected by microscopy. Parasites came back during the third week post-transfection.

### Transgenic lines and parental clone sequencing

To test *PfKelch13* single nucleotide integration, genomic DNA (gDNA) of bulk culture for each transfection (Pf3D7Kelch13^R515K^) was extracted from infected RBCs using the Mini NucleoSpin Blood QuickPure kit (MACHEREY-NAGEL). The high-fidelity polymerase PfuUltra II Fusion HS DNA polymerase was used for PCR amplification to detect the integration of locus. PCR conditions are as follows: 95°C for 2 min, followed by 30 cycles of 95°C for 30 s, 48°C for 20 s, 62°C for 15 s, and a final extension cycle of 72°C for 3 min. PCR products were migrated on 1% agarose gel for 20 min at 100V. NucleoSpin Gel and PCR Clean-up kit (MACHEREY-NAGEL) were used to extract amplified DNA at the expected size. Sequencing of the purified PCR products was done by Eurofins Genomics (TubeSeq service). All primers used in this study are listed in an additional file (**Supplementary Table 1**). Bulk-edited cultures were cloned *via* limiting dilution. For *in vitro* malaria culture synchronous culture of trophozoites stages was used to start the cloning dilution. A 1/1000 dilution was parasitemia checked by microscopy in a 2% hematocrit flask. Parasites were gassed and incubated at 37°C, in a static condition. Genomic DNA from selected clones after serial dilutions of transgenic lines were then amplified and sequence aligned with Pf3D7Kelch13^WT^ parental sequence (**Supplementary Figure 2B**).

### Ring-stage survival assays (RSA^0-3hpi^) investigation of *in vitro* RSA^0-3hpi^ level

*In vitro* RSA^0-3hpi^ were conducted on very early ring-stage parasites (0-3 hours post-invasion; hpi) as previously described (Witkowski et al., 2013). Pf3D7Kelch13^R515K^ clones 1 and 4 were randomly picked and came back from sequencing with correct SNP integration in the Art-r gene marker (**Supplementary Figure 2B**). RSA^0-3hpi^ level of transgenic parasite clones were studied. Parental laboratory strains Pf3D7Kelch13^WT^ and PfNF54Kelch13^C580Y^ were used as negative and positive controls respectively. *In vitro* RSA^0-3hpi^ ring parasites were subjected to sorbitol treatment to eliminate remaining schizonts. Synchronized 0–3hpi rings were next adjusted to 0.5% parasitemia and 2% hematocrit in 1 mL volumes (in 48-well plates), and exposed to DHA (700nM) or 0.1% of its solvent dimethyl sulfoxide (DMSO) as previously described. Duplicate wells were established for each parasite line ± drug. Following wash out, parasites were maintained in complemented drug-free medium in an incubator condition for an additional 66h.

Parasite viability was assessed by microscopic examination of Giemsa-stained thin blood smears by counting viable parasites (**Supplementary File 2C**). A measure of 2 μL of the pellet was then used for each smear. Parasitemia was calculated from a total of at least 10,000 erythrocytes per assay. Slides were read from the two duplicate wells per assay. Percent survival was calculated as the parasitemia in the drug-treated sample divided by the parasitemia in the untreated sample ×100. The assay was done three times to confirm the results. The Mean (SD) of RSA^0-3hpi^ based on three replicates was done.

## Results

### First detection of PfKelch13^R515K^ in *P. falciparum* clinical isolate from Kaolack, Senegal

PfKelch13 polymorphism was studied using the DNA of malaria-infected blood received to investigate a malaria index case reported with a fever persistence upon 3 days ACT treatment (ASAQ) in Kaolack, a low malaria endemic region in Senegal. A total of 15 malaria PfRDT-positive samples were collected including the index case. Sanger sequencing for PfKelch13 was carried out with both a nested PCR and full-length amplification methods. **Table 1a** shows the parasite ART-r gene marker distribution. The index case was infected by parasites carrying a major arginine (AAA coding for K) peak at position 515 of PfKelch13. Both the chromatogram and the msp1 typing confirm a mixed infection with a second clone carrying a low peak of wild-type PfKelch13 (AGA coding for R) (**Figures 1A, B** and **Supplementary Figure 1**). Plasmo typing assay shows 14 out of 15 patients had *P. falciparum* infections and 3 of the 15 had P. *malariae* co-infections (**Supplementary Table 1B**). None of the three remaining human malaria parasites (*P. oval*e, *P. knowlesi*, and *P. vivax*) were detected.

The lack of viable parasites (shipped to IPD a day after sample collection) to be culture-adapted and phenotypically tested was a limiting factor to further investigating ART-r phenotype. We next assessed an *in vitro* phenotypic assay to study the functional relevance of PfKelch13^R515K^.

### Generation of Pf3D7Kelch13^R515K^ CRISPR-Cas9 transgenic lines

**Figure 2:**
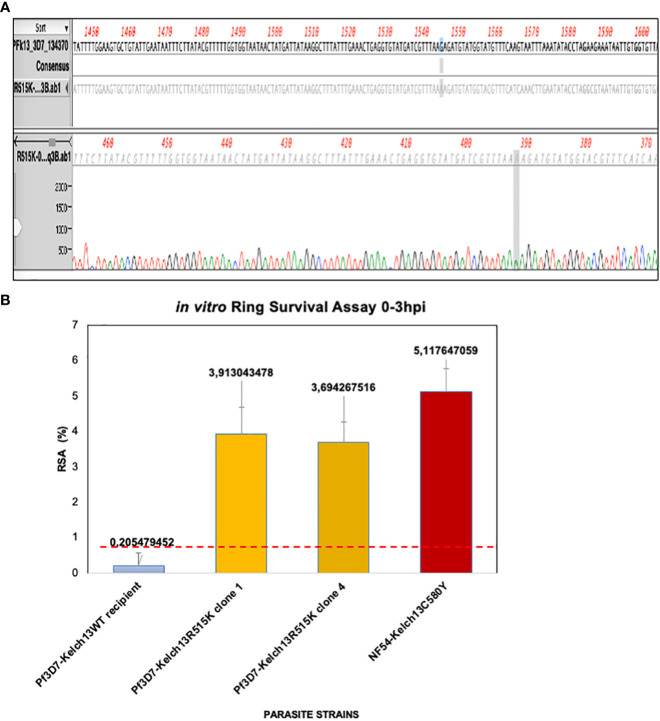
PfKelch13R515K induces increased in vitro RSA0-3hpi value. **(A)** Transgenic lines screening. Sequence confirmation of the generated Pf3D7PfKelch13R515K shows a single nucleotide substitution in the ART-r gene marker when compared to the parental Pf3D7 laboratory line. **(B)** PfKelch13R515K induces increased RSA0-3hpi value. Parasite lines were highly synchronized and grown for one hour under 700nM DHA for 6 hours. Parasitemia was counted by microscopy and RSA level was estimated as survival rate compared to DMSO control lines. The panel shows the level of in vitro ART-r. NF54-Kelch13C580Y shows displaying increased RSA0-3hpi value (5.12%) while Pf3D7Kelch13WT remain sensitive to DHA (0.21%). Two Pf3D7Kelch13R515K clones were used. Error bars of the median of the three biological replicates RSA0-3hpi for each line is shown. *Shows the position of the nucleotide change before and after crispr cas.

pL7-2386R515K plasmid construct was used to generate a transgenic line (**Supplementary Figure 2A**). The resulting bulk cultures were cloned by serial limiting dilution (**Supplementary Figure 2B**). Individual isolated clones were sequenced to verify the creation of the R515K mutation. Pf3D7Kelch13^R515K^ clone sequence is displayed and aligned with its parental Pf3D7 sequence (**Figure 2A**). The most frequent SEA ART-r mutation (Kelch^C580Y^) was used as a positive control line also transfected in an African background *P. falciparum* counterpart PfNF54 (Dondorp et al., 2012; Mbengue et al., 2015; Miotto et al., 2015; Mok et al., 2015; Tun et al., 2015; Bhattacharjee et al., 2018).

### *In vitro* RSA^0-3hpi^ reveals increased Pf3D7Kelch13^R515K^ parasite survival

To validate PfKelch13 R515K mutation as a cause of the fever persistence in the malaria index case reported in Kaolack, we carried out *in vitro* drug sensitivity assay using the standard RSA^0-3hpi^ protocol (Witkowski et al., 2013). Pf3D7Kelch13^R515K^ showed an increased RSA^0-3hpi^ value (**Figure 2B**). Pf3D7Kelch13^R515K^ clones 1 and 4 showed a median RSA of 3.9% and 3.7% respectively while the parental Pf3D7 was sensitive to DHA (median RSA^0-3hpi^ = 0.2%). The parasitemia assessed by microscopy showed a 5.12% RSA^0-3hpi^ value for the positive control NF54Kelch13^C580Y^ (**Supplementary Figure 2C**). In conclusion, our findings demonstrate that 3D7PfKelch13^R515K^ results in an increased *in vitro* RSA^0-3hpi^ value.

## Discussion

The emergence and spread of artemisinin partial resistance in Africa is a major public health concern. There is an urgent need to strengthen ART-r surveillance systems in sub-Saharan Africa. The molecular tool for ART-r provides evidence that PfKelch13 is under selection in African background circulating isolates.

Our finding does show the first appearance of PfKelch13^R515K^ associated with fever persistence upon ACTs treatment in Senegal. PfKelch13^R515K^ mutation is among the mutations shown to be clinically associated with slow parasite clearance. The functional relevance of R515K was studied using an African parasite strain Pf3D7. Pf3D7-Kelch13^R515K^ mutation confers *in vitro* ART-r. Our work is to our knowledge the first PfKelch13^R515K^ *in vitro* phenotypic validation. The presence of R515K mutation raises the possibility that this mutation can be used to assist in the detection of artemisinin partial resistance in Africa. We here determined the power of including genome editing in ART-r molecular tracking and case management in low-middle income countries where sample collection, storage, and transportation to research institutions are often challenging. Our findings suggest a potential risk of discrete artemisinin partial resistance spread across Africa and highlight the need to perform well-designed large-scale surveys.

## Data availability statement

The original contributions presented in the study are included in the article/**Supplementary Material**. Further inquiries can be directed to the corresponding author.

## Ethics statement

The studies involving human participants were reviewed and approved by RÉSEAU 4S Institut Pasteur Dakar. Written informed consent to participate in this study was provided by the participants’ legal guardian/next of kin. Written informed consent was obtained from the individual(s), and minor(s)’ legal guardian/next of kin, for the publication of any potentially identifiable images or data included in this article.

## Author contributions

This manuscript was written by SS, AM. Lab work was done by the G4 unit at Institut Pasteur Dakar, Senegal. SS did the Crispr-cas9 constructs and *in vitro* RSA testings under J-JL-R supervision at University of Montpellier supervised. All authors contributed to the article and approved the submitted version.
