# Supplementary figures and images for: Identification of an in vitro artemisinin-resistant Plasmodium falciparum kelch13 R515K mutant parasite in Senegal
Source: Front Parasitol. 2023 Apr 28;2:1076759. doi: 10.3389/fpara.2023.1076759 (PMC11731788; doi:10.3389/fpara.2023.1076759)

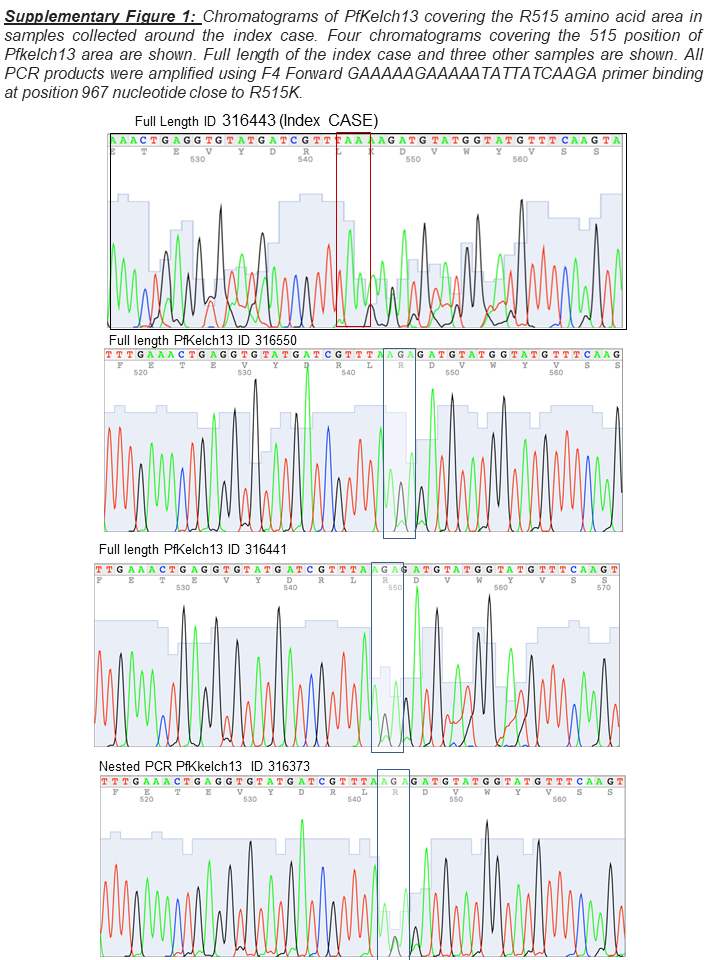

Supplement: Supplementary file 1 [file Image_1.tif]

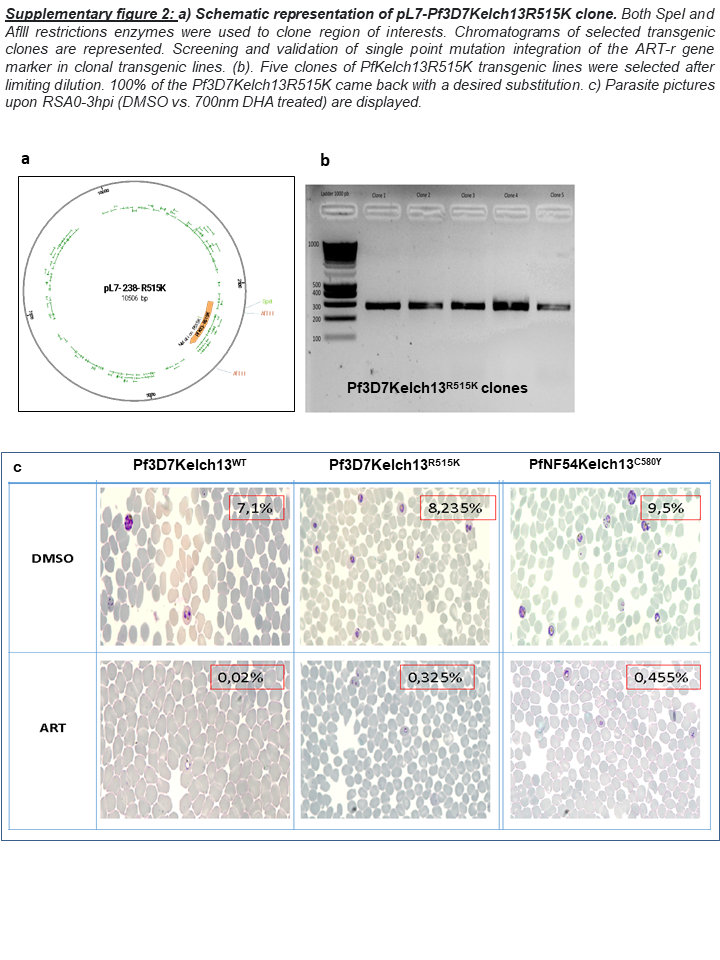

Supplement: Supplementary file 2 [file Image_2.tif]

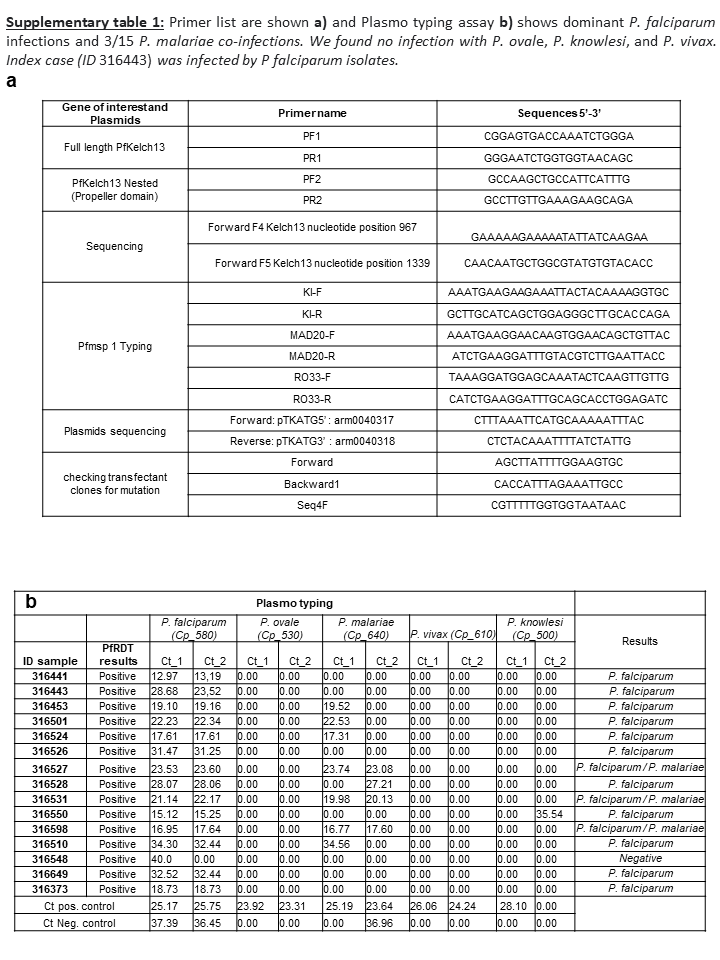

Supplement: Supplementary file 3 [file Image_3.tif]
